# Japanese Patients’ Perceptions of Shared Decision-Making in Renal Replacement Therapy

**DOI:** 10.1016/j.ekir.2025.05.011

**Published:** 2025-05-19

**Authors:** Yugo Shibagaki, Tadashi Sofue, Hiroo Kawarazaki, Tatsunori Toida, Tomo Suzuki, Hiroki Nishiwaki, Kenichiro Asano, Hiroyuki Terawaki, Takafumi Ito, Hideaki Oka, Kei Nagai, Minoru Murakami, Kojiro Nagai, Daisuke Komukai, Takayuki Adachi, Satoshi Furukata, Takaaki Tsutsui, Kiichiro Fujisaki, Seita Sugitani, Hideaki Shimizu, Tomoya Nishino, Hiroaki Asada, Hideki Shimizu, Tatsuo Tsukamoto, Izaya Nakaya, Yosuke Yamada, Ryohei Inanaga, Shohei Yamada, Shohei Nakanishi, Atsuhiro Maeda, Mari Yamamoto, Shuma Hirashio, Takeshi Okamoto, Takayuki Nakamura, Ken-ichi Miyoshi, Hiroshi Kado, Susumu Toda, Shigeru Shibata, Keiko Nishi, Makoto Yamamoto, Tsukasa Naganuma, Ryo Zamami, Masahide Furusho, Hitoshi Miyasato, Yukihiro Tamura, Yoshihiko Raita, Chisato Fukuhara, Keita Uehara, Kosuke Inoue, Yasuhiro Taki, Nobuyuki Nakano, Noriaki Kurita, PREPARES Study Group, Shigeyuki Arai, Tsuyoshi Watanabe, Keita Iwasaki, Yuuki Itou, Fumika Nagase, Kenta Torigoe, Shinichi Abe, Kumiko Muta, Tomomi Endo, Keita Mori, Michiya Shinozaki, Megumi Oikawa, Tsuyoshi Ohshiro, Yoshitaka Ishibashi, Ryo Sugiyama

**Affiliations:** 1 Division of Nephrology and Hypertension, Department of Internal Medicine, St. Marianna University School of Medicine, Kawasaki-city, Kanagawa, Japan; 2 Department of Cardiorenal and Cerebrovascular Medicine, Faculty of Medicine, Kagawa University, Miki-cho, Kita-gun, Kagawa, Japan; 3 Department of Internal Medicine, Division of Nephrology, Teikyo University Hospital Mizonokuchi, Kawasaki-city, Kanagawa, Japan; 4 School of Pharmaceutical Sciences, Kyushu University of Medical Science, Nobeoka-city, Miyazaki, Japan; 5 Department of Clinical Epidemiology, Graduate School of Medicine, Fukushima Medical University, Fukushima-city, Fukushima, Japan; 6 Department of Nephrology, Kameda Medical Center, Kamogawa-city, Chiba, Japan; 7 Division of Nephrology, Department of Internal Medicine, Showa University Fujigaoka Hospital, Yokohama-city, Kanagawa, Japan; 8 Department of Nephrology, Kurashiki Central Hospital, Kurashiki-city, Okayama, Japan; 9 Department of Internal Medicine, Nephrology, Teikyo University Chiba Medical Center, Ichihara-city, Chiba, Japan; 10 Department of Nephrology, Matsuyama Red Cross Hospital, Matsuyama-city, Ehime, Japan; 11 Department of Nephrology, Hitachi General Hospital, Hitachi-city, Ibaraki, Japan; 12 Department of Nephrology, Saku Central Hospital, Saku-city, Nagano, Japan; 13 Department of Nephrology, Shizuoka General Hospital, Shizuoka-city, Shizuoka, Japan; 14 Department of Nephrology, Kawasaki Saiwai Hospital, Kawasaki-city, Kanagawa, Japan; 15 Department of Nephrology, Social Medical Corporation Yuuaikai Yuuai Medical Center, Tomigusuku-city, Okinawa, Japan; 16 Department of Nephrology, Fukaya Red Cross Hospital, Fukaya-city, Saitama, Japan; 17 Kidney Disease and Dialysis Center, Hidaka Hospital, Hidaka-kai, Takasaki-city, Gunma, Japan; 18 Department of Nephrology, Iizuka Hospital, Iizuka-city, Fukuoka, Japan; 19 Department of Nephrology, Japanese Red Cross Wakayama Medical Center, Wakayama-city, Wakayama, Japan; 20 Department of Nephrology, Daido Hospital, Nagoya-city, Aichi, Japan; 21 Department of Nephrology, Nagasaki University Hospital, Nagasaki-city, Nagasaki, Japan; 22 Department of Nephrology, Okazaki City Hospital, Okazaki-city, Aichi, Japan; 23 Department of Nephrology, Funabashi Municipal Medical Center, Funabashi-city, Chiba, Japan; 24 Medical Research Institute Kitano Hospital, PIIF Tazuke-Kofukai, Osaka-city, Osaka, Japan; 25 Department of Nephrology and Rheumatology, Iwate Prefectural Central Hospital, Morioka-city, Iwate, Japan; 26 Department of Nephrology, Aizawa Hospital, Matsumoto-city, Nagano, Japan; 27 Department of Nephrology, Shin-Yurigaoka General Hospital, Kawasaki-city, Kanagawa, Japan; 28 Department of Nephrology, Japanese Red Cross Medical Center, Shibuya-ku, Tokyo, Japan; 29 Department of Nephrology, Hyogo Prefectural Harima-Himeji General Medical Center, Himeji-city, Hyogo, Japan; 30 Department of Nephrology and Dialysis Center, Kouzenkai-Maeda Hospital, Imari-city, Saga, Japan; 31 Department of Rheumatology and Nephrology, Chubu Rosai Hospital, Nagoya-city, Aichi, Japan; 32 Department of Nephrology, NHO Hiroshimanishi Medical Center, Otake-city, Hiroshima, Japan; 33 Division of Nephrology. Nagoya Ekisaikai Hospital, Nagoya-city, Aichi, Japan; 34 Department of Urology, JCHO Nihonmatsu Hospital, Nihonmatsu-city, Fukushima, Japan; 35 Department of Cardiology, Pulmonology, Hypertension and Nephrology, Ehime University Graduate School of Medicine, Toon-city, Ehime, Japan; 36 Department of Nephrology, Omihachiman Community Medical Center, Omihachiman-city, Shiga, Japan; 37 Division of Nephrology, Department of Internal Medicine, Uji Takeda Hospital, Uji-city, Kyoto, Japan; 38 Division of Nephrology, Department of Internal Medicine, Teikyo University School of Medicine, Itabashi-Ku, Tokyo, Japan; 39 Department of Nephrology, Ogawa Clinic, Nobeoka-city, Miyazaki, Japan; 40 Department of Internal Medicine, Okinawa Miyako Hospital, Miyakojima-city, Okinawa, Japan; 41 Department of Nephrology, Yamanashi Prefectural Central Hospital, Kofu-city, Yamanashi, Japan; 42 Department of Cardiovascular Medicine, Nephrology and Neurology, University of the Ryukyus, Nishihara-cho, Okinawa, Japan; 43 Department of Nephrology, National Hospital Organization Kagoshima Medical Center, Kagoshima-city, Kagoshima, Japan; 44 Department of Internal Medicine, Okinawa Prefectural Yaeyama Hospital, Ishigaki-city, Okinawa, Japan; 45 Department of Internal Medicine, Oosumikanoya Hospital, Kanoya-city, Kagoshima, Japan; 46 Department of Nephrology, Okinawa Prefectural Chubu Hospital, Uruma-city, Okinawa, Japan; 47 Department of Nephrology, Heartlife Hospital, Nakagusuku-village, Nakagami-gun, Okinawa; 48 Department of Internal Medicine, Division of Nephrology and Rheumatology, Regional Independent Administrative Corporation Naha City Hospital, Naha-city, Okinawa, Japan; 49 Division of Nephrology, Kochi Memorial Hospital, Kochi-city, Kochi, Japan; 50 Department of Nephrology, Inagi Municipal Hospital, Inagi-city, Tokyo, Japan; 51 Division of Internal Medicine, Clinic of Utsunomiya Jinn-naika-hifuka, Utsunomiya-city, Tochigi, Japan

**Keywords:** chronic kidney disease, health care professionals, patient preferences, renal replacement therapy, shared decision-making

## Abstract

**Introduction:**

Shared decision-making (SDM) is a key process in selecting renal replacement therapy (RRT). This study analyzed SDM perceptions, preferences, as well as patient- and facility-level factors influencing SDM perception among Japanese patients with chronic kidney disease (CKD) who selected RRT.

**Methods:**

We conducted a cross-sectional survey of 475 adult patients with CKD from 49 medical facilities. SDM awareness and recognition, preferences for SDM timing and frequency, discussion content, and desired professional involvement were assessed. Patient- and facility-level factors associated with SDM perceptions were evaluated using multivariable analysis.

**Results:**

The mean participant age was 67.4 years. Hemodialysis, peritoneal dialysis, and kidney transplantation were chosen by 71%, 24.4%, and 4.4% of patients, respectively. Although 81.2% recalled SDM occurring during RRT selection, only 4.7% were well aware of SDM before the survey. Patients prioritized discussions about daily life impact, financial burden, and family-related concerns. Most patients preferred SDM initiation when RRT was imminent, and to be conducted over multiple sessions. Many patients valued the involvement of medical social workers and their usual nonnephrologist physicians in addition to nephrologists. Multiple outpatient visits for RRT selection, involving nurse participation and extended consultation times, were significantly associated with SDM perceptions (prevalence ratio [PR]: 1.59, 95% confidence interval [CI]: 1.05–2.42).

**Conclusion:**

Many Japanese patients with CKD retrospectively evaluated RRT selection as involving SDM; however, a few were familiar with the concept beforehand. This underscores the importance of establishing systems that facilitate repeated SDM discussions at critical moments for patients. These discussions should emphasize the impact of RRT on patients’ lives and involve a multidisciplinary team.

## Graphical abstract

**Figure:**
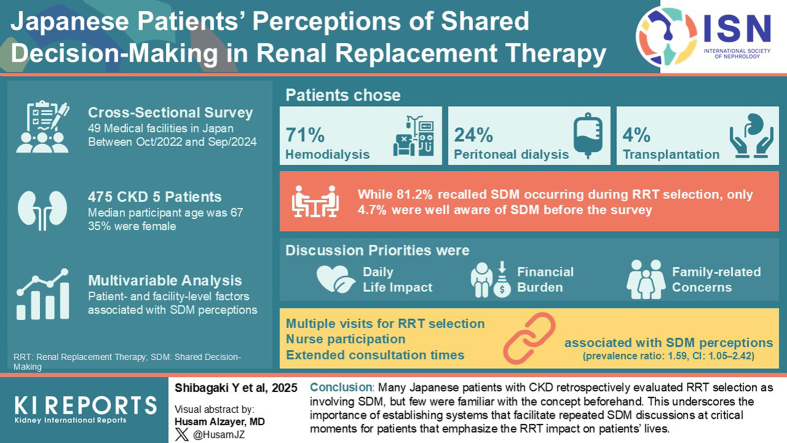

The decision-making process for patients with CKD to select an RRT modality, such as hemodialysis, peritoneal dialysis, or renal transplantation, is a critical issue. A transition from informed choice to SDM has been recommended, because each RRT option is considered a reasonable alternative depending on the patient’s circumstances.^1^ SDM is a collaborative approach in which physicians and patients work together to choose an RRT modality, with the patient contributing expertise on their individual circumstances, values, and goals, and the physician providing expertise on the disease and the risks and benefits of the available treatments.^2^ SDM can reduce regret associated with RRT selection^3^ and may improve treatment adherence,^4^ reduce emergency hospitalizations, and enhance overall prognosis.^5^^,^^6^ However, the optimal timing for initiating SDM, content of discussions, appropriate participants, and factors influencing RRT selection via SDM remain poorly understood.

First, patients with CKD and their physicians face significant challenges in deciding the timing and scope for discussing the choice of RRT.^2^ When kidney failure is not imminent, patients may perceive RRT as a distant concern.^2^ Second, patients often prioritize the impact of RRT on daily life—such as social participation and employment—over life-sustaining benefits and risks associated with the therapy.^7^ Third, decision-making participants may extend beyond the patients themselves and their nephrologists. Although patient autonomy is emphasized in the United States and Europe, with family involvement viewed as either beneficial or detrimental to RRT decision-making^3^^,^^8^; in Asia, including Japan, family involvement is often considered indispensable.^4^^,^^9^ In addition, some patients seek advice from nonnephrologist health care providers with whom they have established long-term relationships.^2^ Fourth, whereas most discussions on promoting SDM focus on patients and health care providers, there is growing recognition of the need to address system-level factors, such as the involvement of multidisciplinary health care teams and the medical reimbursement system.^8^^,^^10^ For example, the Japanese government reimburses outpatient visits for RRT selection if certain facility requirements are met; however, we do not know whether this policy facilitates SDM implementation.

Clarifying these aspects is essential for advancing SDM implementation. Therefore, we conducted a nationwide cross-sectional study involving Japanese adults with CKD who had chosen an RRT modality but had not yet initiated treatment.

## Methods

### Design, Setting, and Participants

This multicenter cross-sectional survey study was conducted between October 2022 and September 2024 at 49 facilities across Japan providing outpatient care for patients with CKD, including dialysis initiation and, at some sites, renal transplantation (Supplementary Figure S1). The study sites included affiliated facilities where alumni of the primary investigator’s department practice, nearby facilities in the Kanto, Shikoku, and Kyushu regions where coinvestigators work, and other facilities with participating nephrologists connected to the research team. These facilities were invited to participate based on personal networks within the nephrology community. The eligibility criteria were as follows: (i) adult patients with stage 5 CKD, (ii) those who had selected one of the modalities of RRT, and (iii) those who had not yet initiated that RRT. For criterion (i), patients were included even if a single measurement of estimated glomerular filtration rate (eGFR) was < 15 ml/min per 1.73 m^2^, provided they also met criteria (ii) and (iii). The exclusion criteria were as follows: (i) patients in whom RRT was emergently initiated before the choice of RRT was made, (ii) patients who were too impaired in physical or cognitive function to complete the questionnaire, or (iii) patients who chose conservative kidney management. The attending physicians consecutively invited eligible patients at these facilities to participate. Using an ethics committee–approved information sheet, they explained the study’s purpose and obtained written informed consent. Patients were rewarded with a 500-yen gift card for completing the questionnaire. The study was approved by the Fukushima Medical University Certified Review Board (No. ippan-2022). The clinical and research activities being reported are consistent with the Principles of the Declaration of Istanbul.

### The Questionnaire Regarding the Choice of RRT and SDM

The self-administered questionnaire, detailed in Supplementary Items S2 to S4, was developed by the study authors, all of whom completed it in a pilot test to refine the items. This questionnaire included items on participant demographics (e.g., education, income, employment status), chosen RRT modality, medical conditions discussed for selection of and preparation for RRT, foundational knowledge on SDM, preferred health care provider involvement in SDM, desired information for SDM, and the timing and frequency of discussion using SDM. The foundational knowledge on SDM included the Introductory Statements for the Questionnaire on SDM (Supplementary Item S3), which concisely defined SDM as a collaborative process in which patients discuss not only medical aspects but also personal values and priorities with health care providers and family members when selecting an RRT option. Both paper and digital versions of the questionnaire were offered, with digital responses enabled via a QR code link on the paper form. The details of the development and administration of the digital version are provided in Supplementary Item S5. Participants were assured that their responses would remain confidential and would not be reviewed by their attending physicians. This policy was upheld through oversight by principal investigators at each site. Responses submitted via paper were directed to a centralized analysis facility for processing and data integration.

### Collecting Clinical and Facility Data

Facility characteristics and clinical data were collected from treating physicians. Patient data included age, sex, primary cause of end-stage kidney disease, frailty (as assessed by the Japanese version of the Clinical Frailty Scale, version 2.0^11^^,^^12^), and the number of outpatient visits for RRT selection. Outpatient visits for RRT selection required the involvement of nursing staff and a minimum consultation time of 30 minutes. However, these visits did not need to be separate from regular nephrology follow-up appointments. In addition, the facility providing these services was not required to meet specific criteria to claim teaching and management fees for RRT under Japan’s unique reimbursement system, which allows up to 2 claims per patient. For example, the facility did not need to have a previous history of claiming reimbursement for peritoneal dialysis teaching and management. Importantly, in facilities that did not meet the criteria for an outpatient visit for RRT selection, nephrologists could have provided repeated explanations about RRT choices within regular nephrology follow-up appointments. However, because these discussions did not meet the predefined requirements (e.g., involvement of nursing staff and a consultation time of at least 30 minutes), they were reported as 0 outpatient visits for RRT selection.

The facility characteristics included the total number of beds, the typical annual volume of RRT initiations, the actual volume of RRT initiations in the most recent quarter, the availability of specialized outpatient visits for RRT selection, the frequency of multiprofessional health care provider involvement in RRT selection discussions, and the types of physicians participating in this process.

### Statistical Analysis

All statistical analyses were performed using Stata/SE version 18 (StataCorp, College Station, TX). For summarizing patient characteristics, facility characteristics, and responses regarding SDM, continuous variables were described as means and SDs, and categorical variables were described as frequencies and percentages. No statistical corrections were made for the nonrepresentative sample.

Sankey diagrams were created to visually represent the distribution of the combined frequency of SDM initiation timing and implementation frequency before RRT selection, as well as the combined frequency of SDM implementation frequency after RRT selection but before initiation, and the willingness to engage in SDM following RRT initiation.^13^

The potential factors associated with strong agreement with having the choice of RRT through the SDM approach were studied via exploratory analysis. The dependent variable was defined based on responses to the question: “Do you feel that your RRTwas chosen through the SDM approach?” with a response of “Strongly agree” (Supplementary Item S4, Question 2). For this, we fit a Poisson regression with cluster-robust variance estimation with facilities as cluster units to estimate the PR of the strong agreement.^14^ Candidate predictors were chosen based on our clinical expertise and forced into the model. For any predictor with missing values, multiple imputation with chained equations was performed assuming that the missing values were at random. Estimates from 10 imputed data were combined into a single estimate.

## Results

### Participant Characteristics

Patient characteristics (*N* = 475) are summarized in Table 1. The mean age was 67.4 years (SD: 13.1), 4 to 5 years younger than the mean age of patients undergoing incident dialysis in Japan (the Japanese Society for Dialysis Therapy [JSDT] registry 2022).^15^ Participants were 65.3% male, with diabetic kidney disease (38.1%), nephrosclerosis (21.1%), and chronic glomerulonephritis (19.8%) noted as the leading causes of end-stage kidney disease, comparable with those of the general Japanese incident dialysis population.^14^ A majority of participants had a high school education or lower (62.1%) and an annual household income < 5 million yen (72.6%). Nearly 60% were retired or unemployed. Approximately 20% of participants were classified as having frailty. Of the participants, 71.0%, 24.4%, and 4.4% selected hemodialysis, peritoneal dialysis, and kidney transplantation, respectively, suggesting a higher rate of nonhemodialysis selection than the national average.^15^ Overall, 44.2% had a single outpatient visit for RRT selection, whereas 30.3% attended ≥ 2 visits.

**Table 1:** Patient characteristics

Patient characteristics, *N* = 475	
Age, mean (SD), yrs	67.4 (13.1)
Male sex, *n* (%)	310 (65.3)
Cause of ESKD, *n* (%)	
Diabetic kidney disease	181 (38.1)
Chronic glomerulonephritis	94 (19.8)
Nephrosclerosis	100 (21.1)
Polycystic kidney disease	23 (4.8)
Others/Unknown	77 (16.2)
Education level, *n* (%)	
Junior high school graduate or less	73 (15.7)
High school graduate	216 (46.4)
University/graduate school graduate	81 (17.4)
Others	96 (20.6)
Missing*, n*	9
Household income, *n* (%)	
< 1 million yen	39 (8.8)
1 million–< 5 million yen	284 (63.8)
5 million–< 10 million yen	86 (19.3)
≥ 10 million yen	36 (8.1)
Missing*, n*	30
Working, *n* (%)	
Yes	181 (38.4)
On leave	14 (3.0)
No	277 (58.7)
Missing*, n*	3
Frailty, *n* (%)	
No frailty	379 (79.8)
Very mild/mild frailty	76 (16.0)
Moderate/severe frailty	20 (4.2)
Very severe frailty/terminally ill	(0)
No. of outpatient visit for RRT selection,^a^*n* (%)	
None	121 (25.5)
Once	210 (44.2)
Twice or more	144 (30.3)
Engagement of primary nephrologist in RRT selection, *n* (%)	
Yes	394 (86.2)
No	63 (13.8)
Missing*, n*	18
Selected RRT, *n* (%)	
Hemodialysis	337 (71.0)
Peritoneal dialysis	116 (24.4)
Kidney transplantation	21 (4.4)
Hemodialysis and peritoneal dialysis	1 (0.2)
Discussions with their physicians treating kidney disease,^b^*n* (%)	
To do or not to pursue RRT	368 (79.3)
Which type of RRT to choose	373 (80.4)
To proceed with or not yet proceed with medical preparation for the selected RRT	359 (77.4)
To start or not yet start the selected RRT in the near future	347 (74.8)
Missing*, n*	11

ESKD, end-stage kidney disease; RRT, renal replacement therapy.

Continuous variables are summarized as the mean with the SD in parentheses, whereas categorical variables are presented as the frequency with the percentage in parentheses.

a Outpatient visits for RRT selection (involving nursing staff collaboration and requiring a minimum of 30 min) were neither required to meet facility criteria for reimbursement claims nor mandated to be separate from regular nephrology outpatient visits.

b Multiple choice allowed.

### Facility Characteristics

The participating facilities varied in bed capacity: 4.1% were clinics, and 8.2%, 18.4%, and 69.4% were low-, low-to-moderate, and moderate-to-high capacity hospitals (Table 2). Specialized outpatient visits for RRT selection were available at 65.3% of the facilities. Regarding multiprofessional participation in explaining RRT options, two-thirds of facilities (67.4%) reported that multiprofessional teams were not involved 70% to 80% of the time. For physician involvement, in 87.8% of the facilities, the patient’s usual physician was responsible for explaining RRT options most of the time (70%–80% of the time).

**Table 2:** Facility characteristics

Facility characteristics, *n* = 49	
Facility type by bed capacity, *n* (%)	
Clinic (No. of beds < 20)	2 (4.1)
Low-capacity hospital (No. of beds 20–< 200)	4 (8.2)
Low-to-moderate–capacity hospital (No. of beds 200–< 400)	9 (18.4)
Moderate-to-high–capacity hospital (No. of beds ≥ 400)	34 (69.4)
Typical annual volume of RRT initiations, *n* (%)	
< 30 cases	15 (30.6)
30–100 cases	26 (53.1)
> 100 cases	8 (16.3)
Actual volume of RRT initiations in the most recent quarter, cases^a^	14.1 (9.3)
Specialized outpatient visit for RRT selection, *n* (%)	
Absent	17 (34.7)
Present	32 (65.3)
Multiprofessional participation in explaining choice of RRT, *n* (%)	
Most of the time (70%–80% of the time), yes	9 (18.4)
Most of the time (70%–80% of the time), no	33 (67.4)
Varies, depending on the case	2 (4.1)
Outpatient service in choice of RRT provided by nurses only	5 (10.2)
Physicians explaining choice of RRT, *n* (%)	
Most of the time (70%–80% of the time), the patient's usual nephrologist	43 (87.8)
Most of the time (70%–80% of the time), the physician in charge of specialized outpatient visits for RRT selection	5 (10.2)
Varies, depending on the case	1 (2.0)

RRT, renal replacement therapy.

Continuous variables are summarized as the mean with the SD in parentheses, whereas categorical variables are presented as the frequency with the percentage in parentheses.

a The median was 12, with a range of 1 to 46.

### Patient Response to Items Regarding SDM

#### Awareness of SDM and Perception of SDM Process for RRT Selection

Familiarity with SDM varied considerably (Table 3). Only a small proportion (4.7%) reported being well aware of SDM, with an additional 18.4% being somewhat aware of the approach. A majority (57.7%) were completely unaware of the approach. A substantial proportion indicated a positive perception regarding SDM process for RRT selection, with 39.1% strongly agreeing and 42.1% somewhat agreeing.

**Table 3:** Patients response to questionnaire items regarding SDM

	*n* (%)
Were you aware of SDM as a way to choose your renal replacement therapy (e.g., dialysis or transplant)?	
I am well aware of it.	22 (4.7)
I am somewhat aware of it.	87 (18.4)
I have heard of it, but I am not very familiar with it.	91 (19.2)
I am not at all aware of it.	273 (57.7)
Missing*, n*	2
Do you feel that your RRT was chosen through the SDM approach?	
Strongly agree	183 (39.1)
Somewhat agree	197 (42.1)
Somewhat disagree	67 (14.3)
Strongly disagree	21 (4.5)
Missing*, n*	7
During SDM, what additional information would you like to ask your health care provider about, beyond the specific treatment methods?^a^	
Daily life limitations with each treatment	317 (66.7)
Economic burden (financial impact) of each treatment	294 (61.9)
Impact of treatment on family and friends	266 (56.0)
Average life expectancy after starting each treatment	258 (54.3)
Impact of treatment on exercise, hobbies, and travel	253 (53.3)
Life expectancy without dialysis or transplantation	223 (47.0)
Ability to continue working after treatment	187 (39.4)
Ability to continue fulfilling household roles with each treatment	170 (35.8)
Impact of treatment on social activities (e.g., socializing, clubs)	127 (26.7)
Would you like your usual kidney doctor to participate in SDM for choosing your RRT?	
Agree	327 (70.0)
Neither agree nor disagree	127 (27.2)
Disagree	13 (2.8)
Missing*, n*	8
Other health care providers you would like to participate in SDM^a^	
Health care providers with expertise in RRT (e.g., nurses, clinical engineers, transplant coordinators)	338 (71.2)
Social workers (medical social workers)	112 (23.6)
Usual doctor (not specialized in kidney care)	90 (19.0)
Primary home care nurse	45 (9.5)
Care manager	37 (7.8)

RRT, renal replacement therapy; SDM, shared decision-making.

a Multiple choice allowed.

#### Additional Information Desired During SDM

Patients expressed interest in discussing a range of factors beyond specific treatment details. These most frequently included daily living restrictions (66.7%) and financial burdens associated with each treatment (61.9%), followed by information regarding the burden on family and friends (56%). In contrast, life expectancy after treatment initiation (54.3%) and life expectancy without dialysis or transplantation (47%) were less frequently cited.

#### Desire for the Participation of Physicians and Other Health Care Providers in SDM

Most patients (70%) wanted their treating physician to participate actively in the SDM. Patients also expressed a desire for various health care professionals knowledgeable about RRT, such as nurses, clinical engineers, and transplant coordinators (71.2%) to participate in SDM. The participation of their usual physicians outside of kidney care were desired by 23.6% of respondents, along with medical social workers (19%), home care nurses (9.5%), and care managers (7.8%).

#### Desired Timing and Frequency of SDM Before RRT Selection

**Figure 1:**
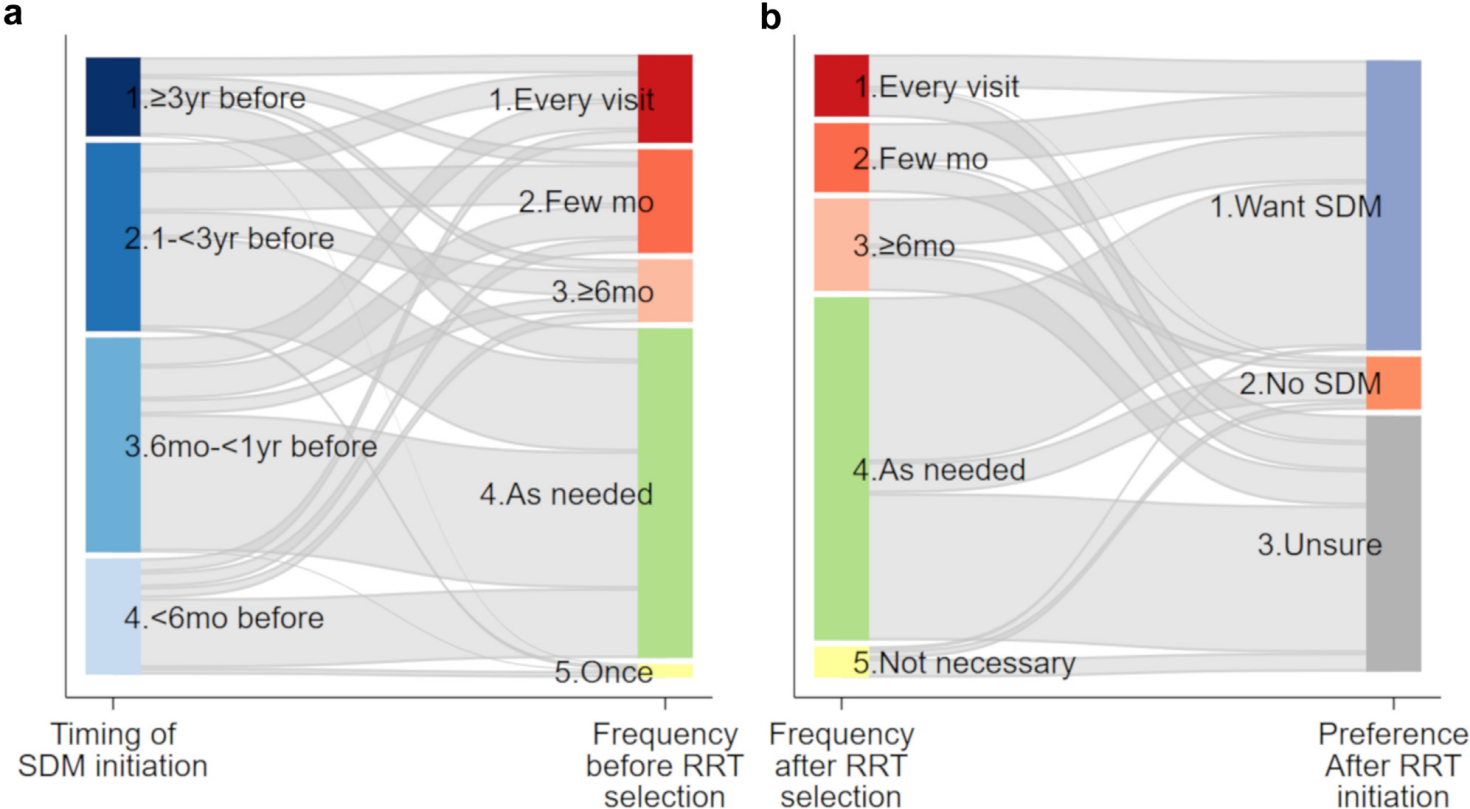
Sankey diagrams. (a) A Sankey diagram illustrating the combination (flow) of the timing and frequency of SDM implementation before RRT selection (*n* = 438). The height of the individual boxes (nodes) on the vertical axis represents relative proportions, whereas the thickness of the links connecting the boxes for timing and frequency reflects the relative proportions of each combination. Deep blue indicates “> 3 years before RRT,” medium blue indicates “1 to < 3 years before RRT,” light blue indicates “6 months to < 1 year before RRT,” and very light blue indicates “> 6 months before RRT.” Red indicates “every visit,” orange indicates “every few months,” light orange indicates “every 6 months or more,” green indicates “as needed,” and light yellow indicates “once." For example, 3.2% of patients reported timing of “> 3 years before” with a frequency of “every visit.” In contrast, 1.1% reported timing of “6 months to < 1 year before” with a frequency of “as needed,” and 22.8% reported timing of “< 6 months before” with a frequency of “once.” (b) A Sankey diagram illustrating the frequency of SDM implementation after RRT selection and preferences for SDM after RRT initiation (*n* = 440). The thickness of the links connecting the boxes for frequency of implementation and preferences after RRT initiation indicates the relative proportions of each combination. Red indicates “every visit,” orange indicates “every few months,” light orange indicates “every 6 months or more,” green indicates “as needed,” and light yellow indicates “not necessary." Blue indicates “want SDM,” orange indicates “no SDM,” and grey indicates “unsure.” RRT, renal replacement therapy; SDM, shared decision-making.

Approximately one-third of patients preferred SDM to start 6 months to 1 year before RRT selection, with another 30% preferring it 1 to 3 years before (Figure 1a). The most common timing-frequency combinations were SDM, 6 months to 1 year before RRT with “as needed” engagement, SDM, 1 to 3 years before RRT with “as needed” engagement, and SDM within 6 months of RRT with “as needed” engagement.

#### Desired Frequency of SDM After RRT Selection and Preference for SDM After RRT Initiation

Over half of the patients preferred “as needed” SDM after RRT selection, while a notable proportion desired regular SDM (“every” or “few months”) (Figure 1b). The most common combination was “as needed” SDM after RRT selection with continued desire for SDM following RRT initiation. The next most frequent combination was “as needed” SDM after RRT selection, followed by uncertainty (unsure) regarding SDM after RRT initiation.

### Factors Associated With a Strong Patient Perception That RRT Selection was Via SDM

Several patient-level characteristics were associated with a strong perception that RRT selection was via SDM (Table 4). Compared with patients with junior high school or lower education, those with a high school education (PR: 0.61, 95% CI: 0.44–0.83), those with a university or graduate school education (PR: 0.64, 95% CI: 0.42–0.95) and those in the "other" education category (PR: 0.75, 95% CI: 0.57–0.996) were less likely to perceive SDM. Patients with an annual income < 1 million yen were less likely to perceive SDM than those with income > 10 million yen (PR: 0.53, 95% CI: 0.32–0.86). Patients with ≥ 2 outpatient visits for RRT selection were more likely to perceive SDM than those with no visits (PR: 1.59, 95% CI: 1.05–2.42).

**Table 4:** Associations of perceived SDM with patients' and facilities' covariates^a^ (*n* = 468)

Characteristics	Adjusted prevalence ratio, point estimate (95% CI)	*P*-value
Patient characteristics		
Age, per 1-yr increase	0.995 (0.986–1.004)	0.266
Men vs. Women	1.08 (0.89–1.31)	0.445
Education level		
Junior high school	Reference	
High school	0.61 (0.44–0.83)	0.002
University/graduate school	0.64 (0.42–0.95)	0.028
Others	0.75 (0.57–0.996)	0.046
Household income		
<1 million yen	0.53 (0.32–0.86)	0.011
1million –< 5 million yen	0.74 (0.51–1.07)	0.112
5 million–< 10 million yen	0.85 (0.55–1.31)	0.462
≥ 10 million yen	Reference	
Working		
Yes	Reference	
On leave	0.82 (0.37–1.81)	0.620
No	1.00 (0.76–1.31)	0.987
Frailty		
Non-frailty	Reference	
Very mild-to-mild frailty	1.10 (0.81–1.49)	0.559
Moderate-to-severe frailty	0.75 (0.37–1.54)	0.436
No. of outpatient visits for RRT selection		
None	Reference	
Once	1.39 (0.96–2.04)	0.085
Twice ore more	1.59 (1.05–2.42)	0.029
Facility characteristics		
Facility type by bed capacity		
Clinic (No. of beds < 20)	Reference	
Low-capacity hospital (No. of beds 20–< 200)	1.79 (0.85–3.76)	0.125
Low-moderate capacity hospital (No. of beds 200–< 400)	1.51 (0.83–2.77)	0.180
Moderate-to-high capacity hospital (No. of beds ≥ 400)	1.99 (1.04–3.78)	0.037
No. of RRT cases initiated over past 1 yr		
< 30 cases	Reference	
30–100 cases	0.65 (0.42–1.01)	0.054
> 100 cases	0.50 (0.30–0.85)	0.011
Availability of specialized outpatient visit for RRT selection	1.17 (0.76–1.82)	0.470
Physicians explaining choice of RRT		
Most of the time (70%–80% of the time), the physician in charge of specialized outpatient visits for RRT selection / vary	Reference	
Most of the time (70%–80% of the time), the patient's usual nephrologist	0.90 (0.61–1.34)	0.606

CI, confidence interval; RRT, renal replacement therapy; SDM, shared decision-making.

a Poisson regression with cluster-robust variance using the facilities as cluster units was fitted, including all variables listed above.

At the facility-level, patients at moderate-to-high–capacity hospitals (≥ 400 beds) were more likely to perceive SDM than those at clinics with < 20 beds (PR: 1.99, 95% CI: 1.04–3.78). However, patients at facilities with > 100 RRT initiations annually were less likely to perceive SDM than those at facilities with < 30 initiations (PR: 0.50, 95% CI: 0.30–0.85). The availability of specialized outpatient visits for RRT selection and the type of physician discussing RRT options with patients were not associated with SDM perceptions.

## Discussion

To the best of our knowledge, this is the first nationwide, multicenter survey in Japan to explore SDM perceptions and preferences for RRT selection among patients with CKD. The survey demonstrated that a high percentage of patients perceive their RRT selection process as involving SDM. It also highlighted key characteristics of the SDM process, including preferred dialogue content, timing and frequency of discussions, desired involvement of specific health care professionals, and system-level factors associated with SDM perceptions.

After being provided with a foundational explanation of SDM as part of this study, over 80% of patients retrospectively perceived their RRT selection process as involving SDM, despite approximately 80% not having a clear understanding of SDM before making their RRT choice. This significant gap underscores a critical issue and emphasizes the need for enhanced communication and education during routine CKD visits.

The fact that most patients prioritize information about how RRT will affect their daily lives—such as limitations on daily activities and the financial burdens associated with treatment—highlights a gap between what patients seek and what health care providers, particularly nephrologists, typically offer.^2^^,^^16^ Although discussing the medical aspects of different RRT modalities and prognoses is undoubtedly important, it represents only one of many concerns for patients in their daily lives.^2^ In fact, for older adults with advanced CKD, maintaining independence often takes precedence over survival.^16^ Although the JSDT proposals emphasize the importance of discussing the impact of RRT on daily life as part of appropriate information provision,^17^ this study suggests that the financial implications should be explicitly included as part of the standard topics for discussion.

Many patients expressed a preference for the timing of SDM to coincide with when RRT became an immediate and tangible concern in their daily lives. Specifically, approximately one-third of patients preferred SDM to occur 6 months to 1 year before RRT selection, whereas about 30% preferred it 1 to 3 years before RRT selection. The JSDT recommends providing information about RRT when eGFR falls below 30 ml/min per 1.73 m^2^.^17^ However, a US study estimated the median time to end-stage kidney disease from an eGFR of 30 ml/min per 1.73 m^2^ to be approximately 6 to 10 years.^18^ A UK qualitative study suggested that decision-making discussions may feel irrelevant to patients who do not anticipate needing to make decisions for several years.^19^ These findings indicate that the timing of SDM may need to be delayed beyond the point recommended by the JSDT, aligning instead with a timeframe that feels more immediately relevant to patients.

The high priority that patients place on discussing the burden on family and friends may reflect the concept of relational autonomy prevalent in Asia, including Japan.^4^ This emphasis not only highlights a cultural context where family members are often actively involved in medical decision-making, but also underscores the patients' own prioritization of burden on family. This prioritization stems from deeply rooted values of family obligations, solidarity, and harmony.^20^ However, there are concerns about complex situations where family involvement may inadvertently lead to decisions that overlook the patient’s true wishes because of implicit pressure.^3^^,^^8^ To address this, as outlined in the JSDT proposal for SDM, it is essential to gather sufficient information about the patient's relationship with their family members.^17^ This ensures a deeper understanding of whether the patient's desire not to burden their family genuinely reflects their own intentions or arises from external pressures.

Several factors may explain why many patients preferred to receive repeated explanations both before and after RRT selection. First, decision-making in RRT is inherently a multistage process.^19^ For example, patients must navigate a series of decisions, starting with whether to undergo RRT, followed by selecting a modality (hemodialysis or peritoneal dialysis), and, if hemodialysis is chosen, determining the type of vascular access (arteriovenous fistula, graft, or catheter). Expecting patients to make these complex decisions in a single outpatient visit is unrealistic. Second, patients may find it difficult to engage in meaningful dialogue about RRT options because of emotional burdens^4^^,^^10^ and hopelessness when confronting the realities of RRT.^4^^,^^21^ The desire for repeated explanations may reflect fluctuating emotions patients experience even after reaching a decision. Third, presenting all the information about RRT in a single, condensed session can overwhelm patients. An Australian qualitative study highlights the importance of iterative discussions, as emphasized by a nephrologist who recognized the need to clarify preferences and confirm decisions over time, while avoiding patient overload.^3^

Finally, it is worth highlighting the association between multiple outpatient visits for RRT selection—featuring multidisciplinary health care provider involvement, including nursing staff, and consultations lasting a minimum of 30 minutes—and enhanced perceptions of SDM. This finding emphasizes the importance of a system-level approach to SDM, incorporating the contributions of various professionals in addition to physicians, particularly in time-constrained settings.^4^^,^^8^ One possible explanation for the observed association between moderate-to-high-capacity hospitals and greater rates of SDM perception may lie in their ability to facilitate more diverse multiprofessional involvement in the RRT decision-making process. For example, in the multiple outpatient visits for RRT selection, nursing staff can provide repeated explanations of RRT options and engage with patients to explore their preferences, values, and needs.^8^ In the moderate-to-high-capacity hospitals, medical social workers can assist in preparing for the SDM process by offering guidance on financial support and access to social services.^8^ The observation that patients perceive SDM more positively during multiple outpatient visits, as opposed to a single visit, aligns with findings in the general population, where more frequent visits are associated with a higher likelihood of recognizing person-centered interactions that include SDM elements.^22^ In addition, given that patients often place greater trust in their usual nonnephrologist physicians with whom they have established longer-term relationships,^23^ incorporating these professionals’ perspectives into RRT selection may prove valuable.^2^ Care continuity provided by multiprofessional health care teams, alongside coordination with patients’ usual nonkidney physicians, should be prioritized to facilitate optimal RRT selection decisions.^8^ In additiony, reimbursement policies should account for these practical and patient-centered care approaches to ensure their sustainability and effectiveness. At the same time, caution may be warranted when extending the potential multidisciplinary actors identified in this study to international contexts, where such teams may be composed of different professionals.^10^^,^^24^ It is important to note that the combined proportion of patients reporting “none” or “once” outpatient visits for RRT selection was 69.7%, but 81.2% agreed or strongly agreed that their RRT choice was made through an SDM approach, which may appear contradictory. However, patients may still perceive a high perception of SDM even without extended consultation time or nursing staff involvement. Because our study examined perceptions of SDM delivery rather than models of delivered SDM, such perceptions could be achieved without formalized, extended consultations with multidisciplinary teams. Because SDM is fundamentally based on physician-patient dialogue, the association between SDM perception and educational level or socioeconomic status in our context aligns with known SDM barriers in CKD.^10^ However, the observed relationship with higher education levels contrasts with existing findings. Although the underlying reason remains unclear, previous studies suggest that highly educated Japanese patients tend to have lower trust in physicians.^25^ Further research is needed to explore whether this is linked to critical health literacy and its impact on SDM perception.^26^

The strength of this study lies in its multicenter design, encompassing 49 medical facilities with small to high bed capacities across Japan, thereby ensuring broad generalizability within the Japanese context. This design also enabled the assessment of the influence of facility-level structural variations on SDM perception.

However, some limitations must be acknowledged. First, the participating facilities may have been skewed toward moderate- to high-bed-capacity hospitals, because a relatively large proportion of included facilities were those with well-qualified nephrologists known to the authors. This selection bias may partially explain the high percentage of patients who perceived their RRT selection process as involving SDM. Such facilities often have specialized outpatient services for RRT selection and provide opportunities for multidisciplinary health care providers to participate, creating ideal conditions for RRT-related SDM. Indeed, 65.3% of the participating facilities met the criteria for specialized outpatient visits for RRT selection, suggesting the integration of specialist nurses and validated decision-aids into these visits. However, the extent to which these elements are implemented in facilities that did not meet the criteria remains unclear, warranting further investigation. Second, we did not examine the number of eligible patients with CKD who declined to participate in the study or those who participated but did not complete the survey. Based on the number of RRT initiations per quarter at each facility and the duration of the survey, we estimate that approximately 1850 RRTs were initiated across the participating facilities. Although this total includes patients who opted out or were ineligible because of emergent RRT initiation or conditions such as dementia, it is noteworthy that patients with CKD who participated in the study represented approximately 26% of all RRT initiations. Third, the study excluded conservative kidney management from its primary focus, and data on the number of such patients were not collected. In Japan, conservative kidney management is increasingly recommended for frail patients or those at high risk of mortality,^17^ underscoring the need for further research in this area. Fourth, SDM perception was assessed via patient self-report rather than actual outpatient records. Thus, even if health care providers presented treatment options, explained their characteristics, and elicited patients' preferences and values, patients might not recall these interactions accurately. However, because our study targeted patients with CKD who had selected an RRT modality but had not yet commenced treatment, we anticipate that most patients could recall the selection process to a reasonable extent. Finally, because of fluctuations in eGFR, it is possible that a small number of patients whose eGFR later increased to > 15 ml/min per 1.73 m^2^ after selecting RRT were included in the study.

In summary, this nationwide study revealed that most patients with CKD in Japan perceive their RRT selection process as involving SDM. Patients prioritize discussions about the effects of RRT on daily life, financial burdens, and the impact on family members over medical aspects. They also express a preference for involving their usual no-nephrology physician and a multidisciplinary health care team in the RRT decision-making process. In addition, patients preferred that SDM take place when RRT initiation became an imminent concern, occurring over multiple sessions rather than a single visit. Notably, patients who attended multiple outpatient visits for RRT selection, with participation from nurses and with sufficient time being allocated for discussion, were more likely to perceive the process as SDM. Future research should explore how the facility characteristics and perceptions of SDM influence actual RRT selection and subsequent outcomes.

## Appendix

### List of the other members of the PREPARES Study Group

Shigeyuki Arai, Tsuyoshi Watanabe, Keita Iwasaki, Yuuki Itou, Fumika Nagase, Kenta Torigoe, Shinichi Abe, Kumiko Muta, Tomomi Endo, Keita Mori, Michiya Shinozaki, Megumi Oikawa, Tsuyoshi Ohshiro, Yoshitaka Ishibashi, and Ryo Sugiyama.

## Disclosure

TSo has received payment for speaking from AstraZeneca K.K., Astellas Pharma Inc., and Kyowa Kirin Co., Ltd. TT has received consulting fees from Astellas Pharma Inc. and payment for speaking and educational events from Torii Pharmaceutical Co., Ltd., Ono Pharmaceutical Co., Ltd., Kyowa Kirin Co., Ltd., AstraZeneca K.K., Nobelpharma Co., Ltd., and Novo Nordisk Pharma Ltd. TSu has received payment for speaking and educational events from Astellas Pharma Inc, AstraZeneca K.K, Vantive Japan, Daiichi Sankyo Co., Ltd., Janssen Pharmaceutical K.K, Kaneka Medix Corp, Kissei Pharmaceutical Co., Ltd., Kowa Co., Ltd., Kyowa Kirin Co., Ltd, Mochida Pharmaceutical Co., Ltd., Nobelpharma Co., Ltd, Novartis Pharma K.K., Novo Nordisk Pharma., Ltd., Ono Pharmaceutical Co., Ltd., Otsuka Pharmaceutical, Terumo Corp, and Torii Pharmaceutical Co., Ltd. HT has received consulting fees from Aplause Pharma, and payment for speaking from Kyowa Kirin Co., Ltd., Mochida Pharmaceutical Co., Ltd., Mitsubishi Tanabe Pharma Corporation, Terumo Corporation, JMS Co., Ltd., Vantive Japan, Torii Pharmaceutical Co., Ltd., Fuji Systems Corporation, and Sanwa Kagaku Kenkyujo Co., Ltd. RI has received payment for speaking and educational events from Vantive Japan. SShibata received personal fees and/or research funding from AstraZeneca, Bayer, Daiichi-Sankyo, Fuji Yakuhin, Kyowa-Kirin, Mochida, and Torii. NK has received consulting fees from GlaxoSmithKline K.K.; and payment for speaking and educational events from Eisai Co., Ltd., Taisho Pharmaceutical Co., Ltd., Kyowa Kirin Co., Ltd., GlaxoSmithKline K.K., Takeda Pharmaceutical Co., Ltd., Kissei Pharmaceutical Co., Ltd., and Baxter Corporation. All the other authors declared no competing interests.

## Supplementary Material

Supplementary File (PDF) **Figure S1.** National map of participating facilities. **Item S1.** Full details of the other members of the PREPARES Study Group. **Item S2.** Questionnaire items regarding the choice of renal replacement therapy. **Item S3.** Introductory statements for the questionnaire on shared decision-making. **Item S4.** Questionnaire items regarding shared decision-making. **Item S5.** Details of online survey development and administration, to provide relevant CHERRIES checklist items. CHERRIES Checklist.

## Footnotes

Supplementary File (PDF)
**Figure S1.** National map of participating facilities. **Item S1.** Full details of the other members of the PREPARES Study Group. **Item S2.** Questionnaire items regarding the choice of renal replacement therapy. **Item S3.** Introductory statements for the questionnaire on shared decision-making. **Item S4.** Questionnaire items regarding shared decision-making. **Item S5.** Details of online survey development and administration, to provide relevant CHERRIES checklist items. CHERRIES Checklist.
