# Supplementary material for: Japanese Patients’ Perceptions of Shared Decision-Making in Renal Replacement Therapy
Source: Kidney Int Rep. 2025 May 19;10(8):2778–88. doi: 10.1016/j.ekir.2025.05.011 (PMC12348115; doi:10.1016/j.ekir.2025.05.011)
Supplement: Supplementary File (PDF) — Figure S1. National map of participating facilities. Item S1. Full details of the other members of the PREPARES Study Group. Item S2. Questionnaire items regarding the choice of renal replacement therapy. Item S3. Introductory statements for the questionnaire on shared decision-making. Item S4. Questionnaire items regarding shared decision-making. Item S5. Details of online survey development and administration, to provide relevant CHERRIES checklist items. CHERRIES Checklist. [file mmc1.pdf]

Supplementary Material

Supplementary Figure S1. National Map of Participating Facilities

The zip codes of participating facilities have been mapped using Google My Maps. To view the distribution on Google My Maps, please click the following link: https://tinyurl.com/mzhhx5vu.

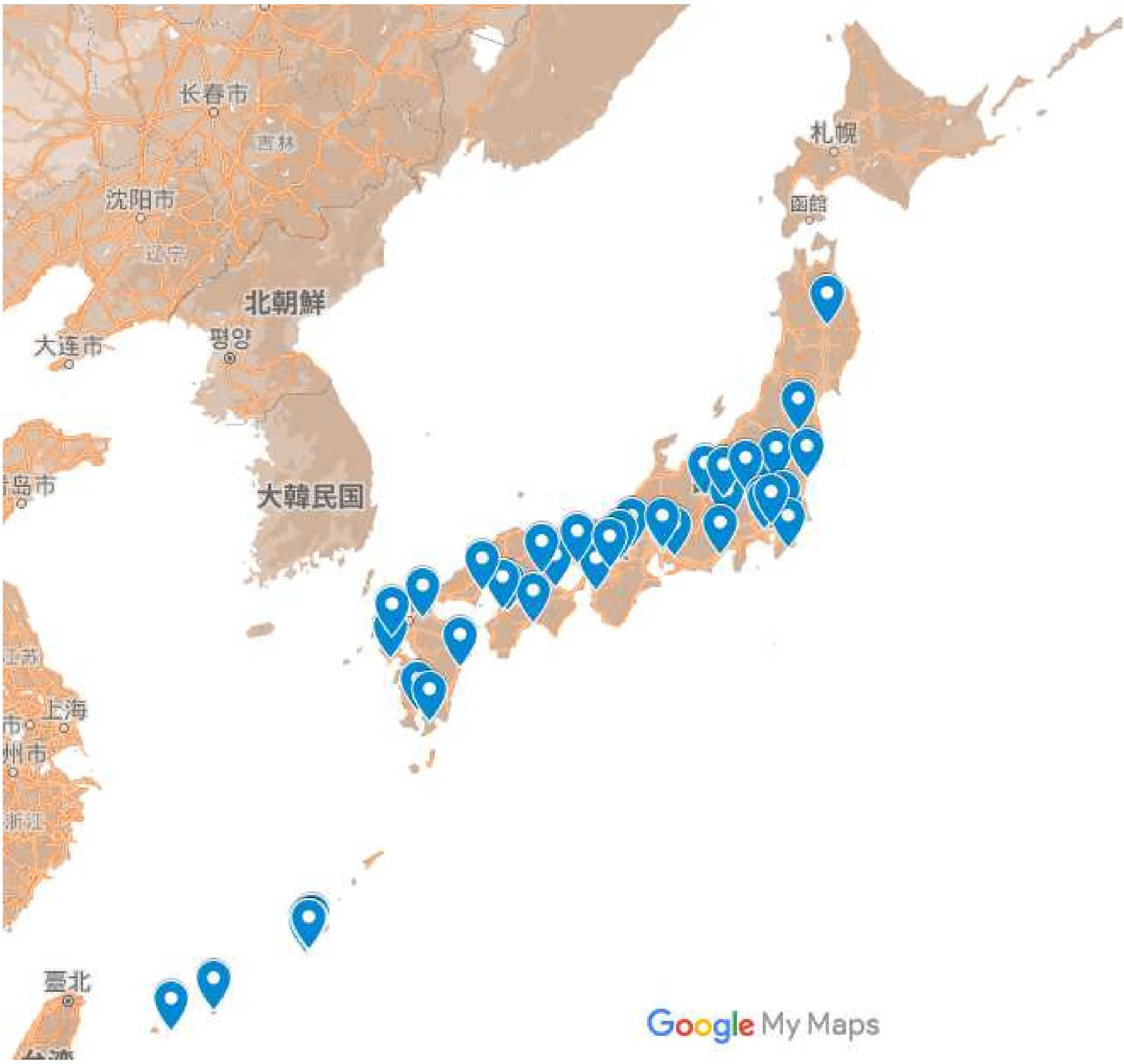

Supplementary Item S1. Full details of the other members of the PREPARES Study Group[†]

[†]The PREPARES Study stands for **PREference for PAtient REnal replacement therapy and Sharing Study**.

Shigeyuki Arai, MD, PhD[1]; Tsuyoshi Watanabe, MD, PhD[2]; Keita Iwasaki, MD[2]; Yuuki Itou, MD[2]; Fumika Nagase, MD[2]; Kenta Torigoe, MD, PhD[3]; Shinichi Abe, MD, PhD[3]; Kumiko Muta, MD, PhD[3]; Tomomi Endo, MD, PhD[4]; Keita Mori, MD, PhD[4]; Michiya Shinozaki, MD, PhD[5]; Megumi Oikawa, MD, PhD[6]; Tsuyoshi Ohshiro, MD[6]; Yoshitaka Ishibashi, MD, PhD[7]; Ryo Sugiyama, MD[8];

[1]Division of Nephrology, Department of Internal Medicine, Teikyo University School of Medicine, Itabashi-Ku, Tokyo, Japan
[2]Department of Rheumatology and Nephrology, Chubu Rosai Hospital, Nagoya-City, Aichi, Japan
[3]Department of Nephrology, Nagasaki University Hospital, Nagasaki-City, Nagasaki, Japan
[4]Medical Research Institute Kitano Hospital, PIIF Tazuke-Kofukai, Osaka-city, Osaka, Japan
[5]Department of Nephrology, Shin-Yurigaoka General Hospital, Kawasaki-City, Kanagawa, Japan
[6]Division of Nephrology, Department of Internal Medicine, Showa University Fujigaoka Hospital, Yokohama-City, Kanagawa, Japan
[7]Department of Nephrology, Japanese Red Cross Medical Center, Shibuya-ku, Tokyo, Japan
[8]Department of internal medicine, Okinawa prefectural Yaeyama Hospital, Ishigaki-city, Okinawa, Japan

Supplementary Item S2. Questionnaire Items Regarding the Choice of Renal Replacement Therapy

1. Was your current primary kidney doctor involved in your choice of renal replacement therapy?

Yes

No

2. Have you and your kidney doctor ever discussed the following? (Please circle all that apply.)

1. Whether or not to pursue renal replacement therapy (dialysis or kidney transplant).
2. Which type of renal replacement therapy to choose (hemodialysis, peritoneal dialysis, or kidney transplant).
3. Whether to proceed with or not yet proceed with medical preparation for the selected renal replacement therapy (such as vascular access surgery for hemodialysis or catheter placement for peritoneal dialysis).
4. Whether to start or not yet start the selected renal replacement therapy in the near future.
5. Other (please specify): _______________

Supplementary Item S3. Introductory Statements for the Questionnaire on Shared Decision-Making

Please read this page carefully before proceeding to the questions on the next page.

Renal replacement therapy options include hemodialysis, peritoneal dialysis, and kidney transplantation, and you will need to choose the one that best suits you.

Choosing the right treatment depends not only on your medical condition but also on your personal values, outlook on life, and lifestyle, including your daily schedule and activities. Therefore, it is important to consider not only the medical suitability of each option but also what matters most to you personally, such as your values, hobbies, preferences, and lifestyle.

We recommend discussing these options with your healthcare providers (e.g., doctors, nurses) and trusted individuals (e.g., family members, close friends) to ensure a comprehensive understanding and to choose the option that feels right for you. This process of collaborative discussion is called Shared Decision-Making (SDM).

SDM differs from a process in which treatment options are chosen based solely on the opinion of healthcare providers or only on the preferences of the patient or family members.

In SDM, everyone shares insights on your living environment, lifestyle, hobbies, preferences, and values, along with considering medical factors, to identify which treatment option is most suitable to you. Through these discussions, healthcare providers and patients work together to reach a consensus on the best treatment plan.

Since deciding on a treatment option can have a significant impact on your life, SDM discussions may take more than one session. Sometimes, it may take several sessions to reach a consensus, or, occasionally, a decision may be postponed if consensus is not reached. Preparing for renal replacement therapy typically requires several months, and often up to six months. Additionally, even if you reach an initial agreement on a treatment, you can always revisit and change your decision later if you change your mind during the course of treatment.

In SDM discussions, the healthcare team may include professionals beyond just your doctor, such as nurses and other specialists. They can often share unique insights regarding the treatment's impact on your life. On your side, you may invite a key person, such as a family member or close friend who understands your life, personality, and values, to help you think through the decision. This can be a great support in considering which treatment option best aligns with your values, lifestyle, and family situation. Ultimately, however, the final decision is yours.

Supplementary Item S4. Questionnaire Items Regarding Shared Decision-Making

1. Were you aware of shared decision-making (SDM) as a way to choose your renal replacement therapy (e.g., dialysis or transplant)? (Please select one.)

1. I am well aware of it.
2. I am somewhat aware of it.
3. I have heard of it, but I am not very familiar with it.
4. I am not at all aware of it.

2. Do you feel that your renal replacement therapy was chosen through the SDM approach? (Please select one.)

1. Strongly agree
2. Somewhat agree
3. Somewhat disagree
4. Strongly disagree

Please answer the following questions as if you were choosing your renal replacement therapy (dialysis or transplantation) through shared decision-making (SDM).

(1) When do you think SDM should begin? (Please select one.)

... before starting renal replacement therapy:

a. More than 5 years before
b. 3 to 5 years before
c. 1 to 3 years before
d. 6 months to 1 year before
e. Less than 6 months before

(2) Would you like your usual kidney doctor to participate in SDM for choosing your renal replacement therapy? (Please select one.)

1. Agree
2. Neither agree nor disagree
3. Disagree

(3) Please select other healthcare providers you would like to participate in SDM. (Please select all that apply.)

1. Healthcare providers with expertise in renal replacement therapy (e.g., nurses, clinical engineers, transplant coordinators)
2. Social workers (medical social workers)
3. Usual doctor (not specialized in kidney care)
4. Primary home care nurse
5. Care manager
6. Others (please specify): ______

(4) During SDM, what additional information would you like to ask your healthcare provider about, beyond the specific treatment methods? (Please select all that apply.)

1. Impact of treatment on family and friends
2. Impact of treatment on social activities (e.g., socializing, clubs)
3. Life expectancy without dialysis or transplantation
4. Ability to continue fulfilling household roles with each treatment
5. Impact of treatment on exercise, hobbies, and travel
6. Ability to continue working after treatment
7. Average life expectancy after starting each treatment
8. Daily life limitations with each treatment
9. Economic burden (financial impact) of each treatment

(5) The choice of renal replacement therapy can significantly impact your life, so we will dedicate time during the shared decision-making (SDM) process to discuss it. This discussion can happen more than once. How often should SDM discussions occur? (Please select one.)

a. About once a year

b. About once every six months

c. Once every few months

d. Every visit

e. As needed

f. Just once

g. Other (please specify): ______

(6) Even after you have chosen renal replacement therapy, you may want to change your treatment due to changes in your physical condition or feelings. In that case, you can still modify your treatment. After you and your healthcare provider have selected your renal replacement therapy, how often should we check in to see if you would like to change your treatment? (Please select one.)

a. About once a year

b. About once every six months

c. Once every few months

d. Every visit

e. As needed

f. Not necessary

g. Other (please specify): ______

(7) Even after starting renal replacement therapy, it may be possible to change to other treatment methods. What are your thoughts on performing shared decision-making (SDM) for this purpose? (Please select one)

1. I would like to have SDM after starting renal replacement therapy.
2. I do not need SDM after starting renal replacement therapy.
3. I am unsure.

Supplementary Item S5. Details of Online Survey Development and Administration, to provide relevant CHERRIES checklist items

Data protection

We conducted our online survey using CREATIVE SURVEY, an online data entry platform provided by Creative Survey Inc. (https://jp.creativesurvey.com/). The company is certified in Japan for implementing appropriate personal information protection measures and is also accredited for compliance with the international security standard ISO 27001. These safeguards minimize the risk of unauthorized access.

Development and testing

Before fielding the survey, its usability and technical functionality were tested by the survey design collaborators (Wakita Takafumi and Takako Maeshibu, Kansai University) and one of the authors (Noriaki Kurita).

Open survey versus closed survey

As only consenting patients received a unique web survey link, converted into a QR code from their physician, this study qualifies as a closed survey.

Web/E-mail

For patients opting to respond via an online form, they accessed the survey by scanning a QR code linked to a unique URL issued for each study ID. Responses were directly stored on a secure server, preventing any modifications after submission.

Context

Only eligible patients received a printed QR code linked to a unique survey URL from their physician, ensuring no access by others. To minimize selection bias due to varying internet access habits, patients unable to access the online survey were offered a paper-based questionnaire.

Mandatory/voluntary

Only patients who consented to participate were asked to complete either the online survey or a paper questionnaire. Those who preferred the online survey filled it out. Therefore, we consider this a mandatory survey within the study process.

Time/Date

Patients provided consent after receiving a survey request from their physician and could complete the survey at any time without a time limit. However, as they had stage 5 chronic kidney disease and required regular visits, physicians checked survey completion at the next visit, typically within one to two months.

Randomization of items or questionnaires

The questionnaire included printed basic information on SDM, which patients read before answering multiple related items. Therefore, neither the question order nor the response options were randomized.

Number of Items

In principle, one question was displayed per page in the online survey.

Number of screens (pages)

The number of questions and instructions reported in this study corresponded to 18 pages.

Completeness check

No consistency or completeness checks were performed before or after the survey submission.

Review step

Respondents were allowed to change their answers using the back button, but no review step was provided.

Unique site visitor

Patients scanned a QR code linked to a unique URL to access and complete the online questionnaire. As responses were automatically collected for each patient, all data are unique.

View rate (Ratio of unique survey visitors/unique site visitors)

Since each eligible patient was asked to scan a QR code linked to a unique URL to visit the website, the number of website visitors theoretically equals the number of survey visitors (i.e., a 100% view rate). However, the actual number of patients who accessed the website after consenting to participate was not collected, so the exact number is unknown.

Participation rate (Ratio of unique visitors who agreed to participate/unique first survey page visitors)

Only patients who consented to participate after receiving an explanation from their physician were given a QR code linked to a unique URL. Therefore, the number of website visitors theoretically equals the number of patients who agreed to participate (i.e., a 100% participation rate). However, the actual number of patients who consented was not collected, so the exact figure is unknown.

Completion rate (Ratio of users who finished the survey/users who agreed to participate)

The number of patients who completed the online survey was recorded, but the number of patients who agreed to participate after receiving an explanation from their physician was not collected. Therefore, the completion rate is unknown.

Cookies used

The platform used cookies to allow respondents to resume their survey from where they left off if the browser was closed. The cookie was valid for one week.

IP check

The platform identified duplicate entries using cookies and prevented second submissions. Therefore, IP checks were not used.

Log file analysis

The platform identified duplicate entries using cookies and prevented second submissions. Therefore, log file analysis was not required.

Registration

Eligible patients accessed the online survey through a unique URL provided via a QR code. Once the survey was submitted, a second entry was not allowed, making it a "closed" survey.

Handling of incomplete questionnaires

Only completed questionnaires were used for analysis.

Questionnaires submitted with an atypical timestamp

No cut-off point for response time was set.

Checklist for Reporting Results of Internet E-Surveys (CHERRIES)

Checklist Item	***Explanation***	***Page Number***
Describe survey design	Describe target population, sample frame. Is the sample a convenience sample? (In “open” surveys this is most likely.)	11
IRB approval	Mention whether the study has been approved by an IRB.	11
Informed consent	Describe the informed consent process. Where were the participants told the length of time of the survey, which data were stored and where and for how long, who the investigator was, and the purpose of the study?	11
Data protection	If any personal information was collected or stored, describe what mechanisms were used to protect unauthorized access.	Supplementary Item S5
Development and testing	State how the survey was developed, including whether the usability and technical functionality of the electronic questionnaire had been tested before fielding the questionnaire.	Supplementary Item S5
Open survey versus closed survey	An “open survey” is a survey open for each visitor of a site, while a closed survey is only open to a sample which the investigator knows (password-protected survey).	Supplementary Item S5
Contact mode	Indicate whether or not the initial contact with the potential participants was made on the Internet. (Investigators may also send out questionnaires by mail and allow for Web-based data entry.)	11
Advertising the survey	How/where was the survey announced or advertised? Some examples are offline media (newspapers), or online (mailing lists – If yes, which ones?) or banner ads (Where were these banner ads posted and what did they look like?). It is important to know the wording of the announcement as it will heavily influence who chooses to participate. Ideally the survey announcement should be published as an appendix.	11
Web/E-mail	State the type of e-survey (eg, one posted on a Web site, or one sent out through e-mail). If it is an e-mail survey, were the responses entered manually into a database, or was there an automatic method for capturing responses?	Supplementary Item S5
Context	Describe the Web site (for mailing list/newsgroup) in which the survey was posted. What is the Web site about, who is visiting it, what are visitors normally looking for? Discuss to what degree the content of the Web site could pre-select the sample or influence the results. For example, a survey about vaccination on a anti-immunization Web site will have different results from a Web survey conducted on a government Web site	Supplementary Item S5
Mandatory/voluntary	Was it a mandatory survey to be filled in by every visitor who wanted to enter the Web site, or was it a voluntary survey?	Supplementary Item S5
Incentives	Were any incentives offered (eg, monetary, prizes, or non-monetary incentives such as an offer to provide the survey results)?	11

Time/Date	In what timeframe were the data collected?	Supplementary Item S5
Randomization of items or questionnaires	To prevent biases items can be randomized or alternated.	Supplementary Item S5
Adaptive questioning	Use adaptive questioning (certain items, or only conditionally displayed based on responses to other items) to reduce number and complexity of the questions.	Not applicable
Number of Items	What was the number of questionnaire items per page? The number of items is an important factor for the completion rate.	Supplementary Item S5
Number of screens (pages)	Over how many pages was the questionnaire distributed? The number of items is an important factor for the completion rate.	Supplementary Item S5
Completeness check	It is technically possible to do consistency or completeness checks before the questionnaire is submitted. Was this done, and if "yes", how (usually JAVAScript)? An alternative is to check for completeness after the questionnaire has been submitted (and highlight mandatory items). If this has been done, it should be reported. All items should provide a non-response option such as "not applicable" or "rather not say", and selection of one response option should be enforced.	Supplementary Item S5
Review step	State whether respondents were able to review and change their answers (eg, through a Back button or a Review step which displays a summary of the responses and asks the respondents if they are correct).	Supplementary Item S5
Unique site visitor	If you provide view rates or participation rates, you need to define how you determined a unique visitor. There are different techniques available, based on IP addresses or cookies or both.	Supplementary Item S5
View rate (Ratio of unique survey visitors/unique site visitors)	Requires counting unique visitors to the first page of the survey, divided by the number of unique site visitors (not page views!). It is not unusual to have view rates of less than 0.1 % if the survey is voluntary.	Supplementary Item S5
Participation rate (Ratio of unique visitors who agreed to participate/unique first survey page visitors)	Count the unique number of people who filled in the first survey page (or agreed to participate, for example by checking a checkbox), divided by visitors who visit the first page of the survey (or the informed consents page, if present). This can also be called "recruitment" rate.	Supplementary Item S5
Completion rate (Ratio of users who finished the	The number of people submitting the last questionnaire page, divided by the number of people who agreed to participate (or submitted the first survey page). This is only relevant if there is a separate "informed consent" page or if the survey goes over several pages. This is a measure for attrition. Note that	Supplementary Item S5

survey/users who agreed to participate)	"completion" can involve leaving questionnaire items blank. This is not a measure for how completely questionnaires were filled in. (If you need a measure for this, use the word "completeness rate".)	
Cookies used	Indicate whether cookies were used to assign a unique user identifier to each client computer. If so, mention the page on which the cookie was set and read, and how long the cookie was valid. Were duplicate entries avoided by preventing users access to the survey twice; or were duplicate database entries having the same user ID eliminated before analysis? In the latter case, which entries were kept for analysis (eg, the first entry or the most recent)?	Supplementary Item S5
IP check	Indicate whether the IP address of the client computer was used to identify potential duplicate entries from the same user. If so, mention the period of time for which no two entries from the same IP address were allowed (eg, 24 hours). Were duplicate entries avoided by preventing users with the same IP address access to the survey twice; or were duplicate database entries having the same IP address within a given period of time eliminated before analysis? If the latter, which entries were kept for analysis (eg, the first entry or the most recent)?	Supplementary Item S5
Log file analysis	Indicate whether other techniques to analyze the log file for identification of multiple entries were used. If so, please describe.	Supplementary Item S5
Registration	In "closed" (non-open) surveys, users need to login first and it is easier to prevent duplicate entries from the same user. Describe how this was done. For example, was the survey never displayed a second time once the user had filled it in, or was the username stored together with the survey results and later eliminated? If the latter, which entries were kept for analysis (eg, the first entry or the most recent)?	Supplementary Item S5
Handling of incomplete questionnaires	Were only completed questionnaires analyzed? Were questionnaires which terminated early (where, for example, users did not go through all questionnaire pages) also analyzed?	Supplementary Item S5
Questionnaires submitted with an atypical timestamp	Some investigators may measure the time people needed to fill in a questionnaire and exclude questionnaires that were submitted too soon. Specify the timeframe that was used as a cut-off point, and describe how this point was determined.	Supplementary Item S5
Statistical correction	Indicate whether any methods such as weighting of items or propensity scores have been used to adjust for the non-representative sample; if so, please describe the methods.	13

This checklist has been modified from Eysenbach G. Improving the quality of Web surveys: the Checklist for Reporting Results of Internet E-Surveys (CHERRIES). J Med Internet Res. 2004 Sep 29;6(3):e34 [erratum in J Med Internet Res. 2012; 14(1): e8.]. Article available at

https://www.jmir.org/2004/3/e34/; erratum available https://www.jmir.org/2012/1/e8/. Copyright ©Gunther Eysenbach. Originally published in the Journal of Medical Internet Research, 29.9.2004 and 04.01.2012.

This is an open-access article distributed under the terms of the Creative Commons Attribution License (https://creativecommons.org/licenses/by/2.0/), which permits unrestricted use, distribution, and reproduction in any medium, provided the original work, first published in the Journal of Medical Internet Research, is properly cited.
